# Gold nanoparticle-modified screen-printed carbon electrodes for label-free detection of SARS-CoV-2 RNA using drop casting and spray coating methods

**DOI:** 10.5599/admet.2577

**Published:** 2025-02-17

**Authors:** Salma Nur Zakiyyah, Nadya Putri Satriana, Natasha Fransisca, Shabarni Gaffar, Norman Syakir, Irkham, Yeni Wahyuni Hartati

**Affiliations:** 1 Department of Chemistry, Faculty of Mathematics and Natural Sciences, Universitas Padjadjaran, Bandung 40173, Indonesia; 2 Department of Physics, Faculty of Mathematics and Natural Sciences, Universitas Padjadjaran, Bandung 40173, Indonesia

**Keywords:** Electrochemistry, biosensor, drop casting, spray coating, SARS-CoV-2, gold nanoparticles

## Abstract

**Background and purpose:**

This study aimed to explore the modification of screen-printed carbon electrode (SPCE) to produce an extensive conductive surface with gold nanoparticles (AuNPs) for the detection of severe acute respiratory syndrome coronavirus 2 (SARS-CoV-2) ribonucleic acid (RNA).

**Experimental approach:**

The experiment was carried out using drop casting (DC) and spray coating (SC) methods. Au-S covalent interactions were formed between thiolated single-stranded DNA (ssDNA) and Au surface, which further hybridized with the target RNA to be detected using differential pulse voltammetry (DPV). Optimization of experimental conditions was performed using Box-Behnken design (BBD) on probe ssDNA concentration, probe ssDNA immobilization time, and target hybridization time. The morphology of the modified electrode was characterized using a scanning electron microscope, while the electrochemical behaviour was determined with DPV and electron impedance spectroscopy.

**Key results:**

The results showed that SPCE modification with AuNPs by DC produced a higher peak current height of 12.267 μA with an *R*_ct_ value of 2.534 kΩ, while SC improved the distribution of AuNPs in the electrode surface. The optimum experimental conditions obtained using BBD were 0.5 μg mL^-1^ ssDNA-probe concentration, an immobilization time of 22 minutes, and a hybridization time of 12 minutes. The limit of SARS-CoV-2 RNA detection at a concentration range of 0.5 to 10 μg mL^-1^ was 0.1664 and 0.694 μg mL^-1^ for DC and SC, respectively. The T-test results for both methods show that the current response of target RNA with SPCE/AuNP by DC does not show the same result, indicating a significant difference in the current response between those two methods.

**Conclusion:**

SPCE/AuNP by DC is better than SPCE/AuNP by SC for immobilizing inosine-substituted ssDNA, which subsequently hybridizes with viral RNA, enabling label-free detection of guanine from SARS-CoV-2 RNA.

## Introduction

Developments in nanotechnology are significantly contributing to advances in screen-printed carbon electrodes (SPCE) modified with nanomaterials. Generally, there are three electrodes in SPCE, namely carbon-based working, counter, and auxiliary, which serve as promising materials for sensor development that allow fast analysis due to the ability to be miniaturized with a small sample size [1]. The common nanoparticles used for SPCE modification include silver, platinum, metal oxides, carbon nanotubes, and gold. Among those, gold nanoparticles (AuNPs) have the advantage of enhancing conductivity and catalytic properties [2] while the same has good compatibility with biomolecules. SPCE modified with AuNPs has been widely produced and provides sensitive response results due to its high electrical conductivity. In electrochemical biosensing, AuNPs can form strong Au-S bonds in self-assembly monolayer (SAMs) patterns, efficiently immobilizing thiolated probes [3-5]. Therefore, AuNPs can improve electroanalytical performance better than bare electrodes due to the ability to increase conductivity, sensitivity, detection limits, and stability [6-8].

The modification of the electrode requires careful consideration to avoid changing the chemical integrity during the expansion of the surface area [9]. In this context, SPCE modification is carried out using various methods such as electrodeposition [10], drop casting (DC) [11,12], spin coating [13], layer-by-layer assembly (LbL) [14], dip coating [15], and spray coating (SC) [16]. The most common methods are electrodeposition, DC, and SC methods due to their advantages as they provide stability and homogeneity on the SPCE surface, while DC and SC are considered the simplest and easiest methods for modification using nanoparticles [17,18].

DC method is an electrode modification that is often used for a real sample measurement of electrochemical biosensors, including human papillomavirus 18 (HPV-18) [19], RBD protein S SARS-CoV-2 [20,21], SARS-CoV-2 antigen [22], and SARS-CoV-2 antibodies [23]. Mahmoodi *et al.* explored the use of the DC method by dripping the material solution on the electrode surface at a consistent volume, dwell time, and drying [19]. On the other hand, electrochemical biosensors using electrodes modified by SC are still being developed. SC is efficient on an industrial scale due to easy implementation on the electrode surface and the potential use of less volume per electrode [24]. The research reported by Chomoucka *et al.* [25] used carbon paste modified by SC Cu_2_O nanoparticles for the detection of purine bases. During the experiment, spraying carried out approximately 10 times was ineffective because the observed electrode surface showed no difference from the bare surface. This was attributed to the amount of spraying, which affected the thickness and stability of the electrode surface [24,26]. Additionally, SC nanocomposite AuNP-Hydroxyapatite has also been reported [16] to exhibit good sensitivity and performance.

SARS-CoV-2 is an infectious disease in humans caused by the coronavirus. In March 2020, the World Health Organization (WHO) declared this infectious disease a global pandemic due to its rapid transmission worldwide. In the structure of the SAR-CoV-2 virus, there is a single strand of ribonucleic acid (RNA) with four main structural proteins. Taxonomically, SARS-CoV-2 is included in Betacoronavirus (B lineage) and has four genes, namely spike (S), membrane (M), envelope (E), and nucleocapsid (N) [27,28]. Due to the rapid spread, the development of accurate and fast virus detection methods has gained significant attention in recent studies.

Deoxyribonucleic acid (DNA) or RNA-based detection method using electrochemical biosensor on hybridization of probe DNA sequence with its complementary strand has shown high efficiency and specificity [29]. To detect SARS-CoV-2 RNA, RNA-based detection methods can be used due to their specificity, high sensitivity, and good efficiency. However, the selection of probes, reporters, and labels used in RNA biosensors is essential. Hairpin DNA probes have been reported for detecting SARS-CoV-2 RNA by observing Ru(NH_3_)_6_^3+^ [30], methylene blue (MB) and ferrocene (Fc) signals [31]. Another alternative method, namely label-free, has been found to facilitate the detection of electrochemical biosensors [32-35]. Tripathy & Singh [36] reported that the label-free electrochemical biosensor method depended on a nitrogen base oxidation process without several analysis times and was free from steric hindrances. A nitrogen base that is easily oxidized is guanine, showing the need for replacement with a similar structure, such as inosine.

Previous studies explored the development of electrochemical biosensors for SARS-CoV-2 detection using various modification methods such as DC, electrodeposition, and spin coating. For example, the use of AuNPs electrodeposited graphene electrodes for smartphone-based POC sensors produced a value detection limit of 6.9 copies μL^-1^ [37], while Chaibun *et al.* [38] applied SPCE electrodeposited with AuNPs and produced a detection limit value of 1 copies μL^-1^. The study by Fabiani *et al.* [39] used thin-film electrodeposited AuNPs and produced a detection limit value of 111 fM [39]. Furthermore, Raziq *et al.* [22] applied SPCE modified with nano carbon black using DC to produce a detection limit of 8 ng mL^-1^. Mojsoska *et al.* [40] used an SPCE modified by graphene by electrodeposition and produced a detection limit of 20 μg mL^-1^. On the other hand, Rahmati *et al.* [41] applied a screen-printed carbon electrode modified by Cu_2_O nanocubes using spin coating and produced a detection limit value of 0.04 fg mL^-1^. The modification of SPCE by AuNPs was also carried out using DC, with the results showing a detection limit value of 79.79 ng mL^-1^. This study aimed to explore the detection of viral RNA SARS-CoV-2 using SPCE modified with AuNPs through DC and SC methods. The study includes morphology observation using SEM and electrochemical characterization, such as DPV and EIS, for each modification method, as well as experimental design and optimization. This combination of guanine-inosine substitution, SAM-based probe immobilization, and the use of dual modification techniques for SPCE/AuNP preparation represents a novel advancement and its potential for the development of label-free SARS-CoV-2 RNA detection. The findings contribute to the development of reliable, sensitive, and scalable diagnostic tools, particularly for clinical applications in the detection of SARS-CoV-2 viral RNA.

## Experimental

### Materials

The materials used were DNA probe-thiol (5’ACAATTTICCCCCAICITTAI, Bioneer), RNA Target (5’CUAACG--CUGGGGGCAAAUUGU, Bioneer), SPCE consists of carbon-based working (diameter of 2 mm), auxiliary electrodes, and Ag/AgCl as a reference electrode (SPCE, GSI Technologies, USA). Other materials included aquademineralized (aqua-dm, PT Ikapharmindo Putramas, Jakarta), chloroauric acid (HAuCl_4_.3H_2_O, synthesis results from the Chemical Analysis and Separation Laboratory in 2020), sulfuric acid (H_2_SO_4_, Merck, Germany), saline-sodium citrate buffer (SSC, Merck, Germany) pH 7,0, and sodium dodecyl sulphate (SDS). Furthermore, potassium ferricyanide (K_3_[Fe(CN)_6_], Merck, Germany), potassium chloride (KCl, Merck, Germany), sodium hydroxide (NaOH, Merck, Germany), nuclease-free water (NFW, Merck, Germany), and tris 2-carboxyethyl phosphine (TCEP, Sigma Aldrich, USA) were used in this study.

The instrument used to characterize SPCE were ZP potentiostat which was connected to a computer using PSTrace 5.8 software (Zimmer & Peacock, UK), rotatory evaporator (BUCHI B-490, Switzerland), scanning electron microscope with energy dispersive X-ray spectroscopy (SEM-EDX, Hitachi SU3500-EDAX Octane Pro, Tokyo, Japan), UV-VIS spectrophotometer (Thermo Scientific), particle size analyzer (Horiba scientific, Japan) microcentrifuge microCL 17R (Thermo Fisher Scientific, Jerman), and autoclave (Medical 2100, Prestige, USA).

### Modification of screen-printed carbon/gold nanoparticle electrode

HAuCl_4_ was synthesized from 1 g of 99.9 % pure gold, dissolved in 30 mL of aqua regia with slow stirring using a magnetic stirrer. The solution was heated to 50 °C, then gradually to 80 °C until the gold dissolved completely. It was further heated to reduce the volume to 20 mL, ensuring the elimination of brown fumes. The resulting solution was transferred to a round-bottom flask and evaporated under vacuum at 60 °C using a rotary evaporator until the solvent was removed. Subsequently, 10 mL of demineralized water was added to the flask, and the solution was re-evaporated to concentrate it. After cooling to room temperature, the flask was stored in a vacuum desiccator until needle-shaped crystals formed. The crystallized HAuCl_4_ salt was weighed using an analytical balance and dissolved in demineralized water to a final volume of 100 mL, resulting in a HAuCl_4_.3H_2_O solution with a concentration of approximately 25.8 mM.

The synthesized HAuCl_4_ was used to prepare AuNPs through a reduction process. A solution containing 1 mL of 1 mM HAuCl_4_ was mixed with 10 mL of demineralized water in a beaker, followed by the addition of 1 mL of 1 % (w/v) trisodium citrate as a reducing agent under continuous stirring at 90 °C. The colour of the solution gradually changed from pale yellow to deep red, indicating the formation of AuNPs. The resulting colloidal solution was cooled to room temperature and stored for further characterization. UV-Vis spectrophotometry analysis of the AuNPs solution revealed a distinct surface plasmon resonance (SPR) peak at 521 nm, confirming the successful synthesis of AuNPs. Particle size analysis (PSA) showed an average particle size of 15 ± 2 nm, indicating the formation of uniformly distributed nanoparticles.

Before each modification, the SPCE was cleaned with aqua-dm and dried at room temperature. For modification of AuNPs using the DC method, the SPCE bare was dripped by 15 μL of AuNPs colloidal solution and was allowed to dry for 24 hours. On the other hand, to modify AuNPs using the SC method, the SPCE was first heated in UV heat for 15 minutes, then was sprayed with AuNPs colloidal solution 10 times with tool pressure of 45 psi and allowed to dry after each spraying. SPCE/AuNPs were rinsed with water after each step of modification and dried at room temperature. The SPCE/AuNPs were characterized using the differential pulse voltammetry (DPV) method with a redox system consisting of 10 mM K_3_[Fe(CN)_6_] in a 100 mM KCl solution. The measurements were performed over a potential range of -0.8 to +0.8 V, with a scan rate of 0.008 V *s^-1^*, an *E*_step_ of 0.005 V, an *E*_pulse_ of 0.05 V, and a *t*_pulse_ of 0.05 s. Additionally, the SPCE was characterized using electrochemical impedance spectroscopy (EIS) and scanning electron microscopy (SEM) to assess its surface properties and electrochemical behaviour.

### Biosensor response to the detection of SARS-CoV-2 synthetic target RNA

**Figure 1.**
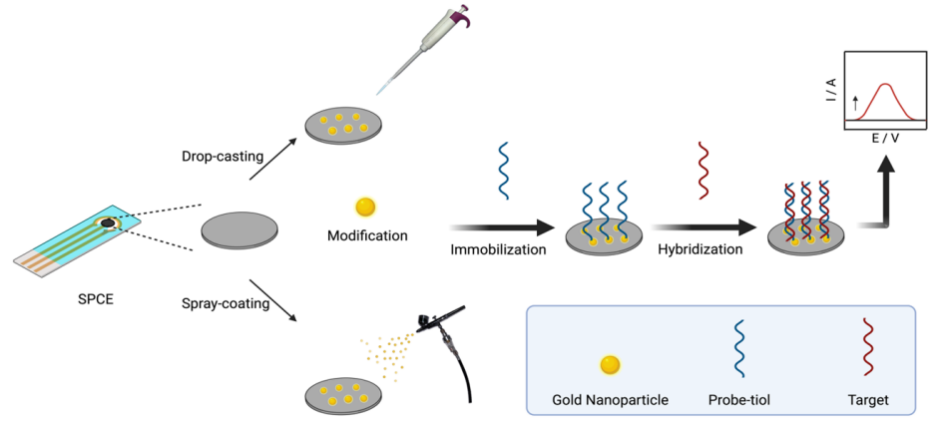
Illustration of SPCE modification with AuNPs using DC and SC techniques of electrochemical DNA biosensor for detection RNA SARS-CoV-2

To prepare the DNA probe solution, 10 μL of a mixture containing the thiolated DNA probe and 5 μL of TCEP was used. Then, 5 μL of the prepared DNA probe solution was applied onto the SPCE/AuNPs surface and incubated for immobilization. After incubation, the SPCE/AuNPs/DNA probe electrodes were thoroughly rinsed five times with SSC buffer solution (pH 7.0) to remove any unbound material. The SPCE/AuNPs/DNA probe was hybridized by applying 5 μL of target RNA onto the electrode surface and incubating it to allow hybridization between the probe and the target. After incubation, the electrode was rinsed five times with a buffer solution composed of SSC buffer and 0.1% SDS (pH 7.0) to remove any unbound target RNA. The guanine oxidation signal was then measured using the DPV technique. The analysis was performed over a potential range of -0.8 to +1.2 V, with a scan rate of 0.008 V s^-1^, an *E*_step_ of 0.005 V, an *E*_pulse_ of 0.05 V, and a *t*_pulse_ of 0.05 s. An illustration of the SPCE modification with AuNPs using the DC and SC techniques is provided in Figure 1.

### Optimization of parameters

The experimental response data were analysed to optimize the instrument parameters and determine the ideal conditions for the system. The Box-Behnken Design (BBD), a statistical approach for response surface methodology (RSM), was employed to systematically evaluate the effects of multiple factors and their interactions. The analysis was conducted using Minitab-18 software, which facilitated the design, execution, and interpretation of the optimization process. Each factor listed in Table 1 was varied according to the BBD framework to generate a set of experimental conditions. The responses were then processed to identify the optimum values for each factor that maximize sensitivity and ensure reproducibility. The optimized parameters were subsequently validated through additional experiments to confirm their effectiveness in enhancing the performance of the system.

**Table 1.** Box-Behnken design for parameter selection

Factor	Level		
	-1	0	+1
Immobilization time, min	10	20	30
DNA probe concentration, μg mL^-1^	0.5	1.0	1.5
Hybridization time, min	5	10	15

### Determining the statistical significance of the T-test

To evaluate the performance of the two modification methods—drop casting and spray coating—the T-test was conducted based on the average current values obtained from the SPCE/AuNPs electrodes. The current measurements were recorded four times for each method using DPV. The mean and standard deviation of the current values were calculated for both methods, providing the necessary data for statistical comparison. The T-test was performed to determine whether the two methods had a statistically significant difference. The test statistic (*t*_count_) was calculated using Equation (1).



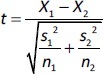

(1)


Where *X*_1_ and *X*_2_ are the mean current values for drop casting and spray coating, respectively; *S*_1_^2^ and *S*_2_^2^ are the variances of the current values for the two methods and *n*_1_ and *n*_2_ are the sample sizes (number of measurements) for each method.

## Results and discussion

### Characterization of screen-printed carbon/gold nanoparticle electrode

The modification of SPCE/AuNPs was conducted using DC and SC methods to increase the electron transfer rate to the electrode surface and the ability to immobilize biomolecules. The mechanism of modification using the DC method included the spreading of nanoparticle solution droplets, leading to extensive coverage on the surface. For optimal adherence of nanoparticles, the AuNP solution was left for 24 hours, and it remained on the electrode surface while the unattached solution was washed away. The quantity of adhered nanoparticles was controlled by the volume, concentration, and time of AuNPs solution under optimal conditions. Before SC, pretreatment of SPCE was performed by exposing samples to ultraviolet light for 15 minutes to oxidize the carbon particles so that the carbon functional groups on the surface of SPCE would remain active. The activation showed that carbon from the relaxation energy level was excited to a higher energy level. This pretreatment was also carried out to clean the electrode surface, change the microstructure and surface chemistry, and produce new active sites affecting the sensitivity of the electrode to the target analyte.

The mechanism of SPCE/AuNPs using the SC method includes the formation of fine aerosols through a nozzle jet during application. Aerosol droplets hit the electrode surface when sprayed and stick to the electrode surface after modification. AuNPs spread evenly across the entire electrode surface, producing a uniform placement of nanoparticles similar to a film layer. However, several factors, such as volume, should be considered. This is because a larger volume is essential, as some spraying that does not precisely hit the electrode surface will be wasted. After spraying, the AuNPs solution is left on the electrode surface until it dries for proper adherence.

**Figure 2.**
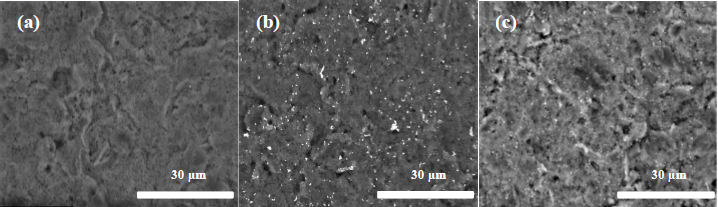
Images of morphological surface observation using SEM for (a) bare SPCE; (b) SPCE/AuNP by DC; and (c) SPCE/AuNP by SC

SEM characterization results of the SPCE surface are shown in Figure 2. Specifically, Figure 2a presents the SPCE bare surface before modification, while Figures 2b and 2c show modified SPCE/AuNPs using DC and SC, respectively. DC method shows a higher quantity of nanoparticles than SC, which has a more homogeneous distribution.

**Figure 3.**
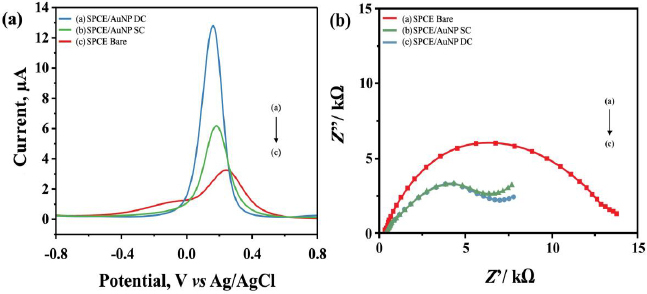
(a) DPV voltammogram of SPCE in scan rate of 0.008 V s^*-1*^ in the potential range of -0.8 to 0.8 V with a scan rate of 0.008 V s^*-1*^, *E*_step_ 0.005 V, *E*_pulse_ 0.05 V, and *t*_pulse_ 0.05 s and (b) Nyquist plot of SPCE in a frequency of 0.1 to 1000000 Hz at anodic peak current potential of 0.01 V using 10 mM K_3_[Fe(CN)_6_] 10 mM in 100 mM KCl solution

Figure 3a shows DPV voltammograms of SPCE/AuNP before and after modification using DC and SC in the K_3_[Fe(CN)_6_] redox system. Curves a and b show the increase in the peak current response of SPCE/AuNPs. Based on the results, the modification process shows increased conductivity and electron transfer in SPCE/AuNP compared to bare SPCE. Based on the result, both modification processes show an increase in peak current, suggesting higher conductivities and higher electron transfer rates for oxidation of the iron cyanide. In comparison, the DC method produces a more significant increase in peak current of 12.267 μA (Figure 3a, blue line) than the SC method at 6.179 μA (Figure 3a, green line).

The consistency of concentration in a fixed volume can be a significant factor in the modification of SPCE/AuNPs, although it is less homogeneous compared to the SC method (Figure 2). The results suggest that the advantage of DC includes the requirement of large volume along with the ability to maintain consistency and stability, while SC offers better uniformity but requires further optimization to increase the current to a higher level by adjusting the distance, volume, spraying rate, and time.

The EIS characterization results were observed from the semicircular part of the Nyquist plot at higher frequencies related to the electron transfer process. As the current increases due to electron transfer, the impedance and *R*_ct_ diameter will become smaller [42]. The comparison of *R*_ct_ values before and after modification of AuNPs is shown in Figure 3b. The *R*_ct_ diameter for the bare SPCE (Figure 3b green line, 4.543 kΩ) decreases significantly compared to the diameter of SPCE/AuNP both via SC (Figure 3b purple line, 2.698 kΩ) and DC (Figure 3b red line, 2.698 kΩ). The large surface area of AuNPs, electron transfer properties and electrocatalytic activity contribute to a significant reduction in resistance, thereby accelerating the electron transfer rate [43].

### Electrochemical biosensor for the detection of SARS-CoV-2 RNA and its morphology

In this study, the modification of SPCE/AuNPs was carried out using DC and SC methods for biosensors to detect SARS-CoV-2. These biosensors depended on SPCE/AuNPs for direct hybridization to detect the target RNA sequence. The intrinsic signal of the targeted guanine was applied without using external redox indicators depending on nitrogen base oxidation processes. To ensure accurate detection, guanine in the probe was converted into inosine.

The thiolated DNA probe was mixed with TCEP before being dropped onto SPCE/AuNPs. TCEP was a potent thiol-free reducing agent that was stable and soluble in many aqueous solutions at any pH. Since the initial thiolated ssDNA probe lacked a free thiol at the 5' end, thiol was capped with a protective disulfide (S-S) bond. TCEP would remove protective disulfide bonds in thiol-modified ssDNA probes. Therefore, a free thiol (-SH) modified ssDNA probe could interact with Au [44].

Immobilization of the thiolated DNA probe was performed on the electrode surface through a self-assembled monolayer (SAM) with an Au surface, forming strong affinity interactions to create an S-Au covalent bond, which produced a dense and organized monolayer [45]. S-Au reaction was a Lewis acid-base, where the free electrons on S atoms bind with Au. Subsequently, sulphur atoms became positively charged and released hydrogen, making S neutral (tending to be stable). Au captures electrons from S, converting Au^0^ to Au^-^. To stabilize Au^-^ back to Au^0^, electrons from Au^-^ are used to reduce H^+^ to H_2_, forming SAM between Au and S [46].

**Figure 4.**
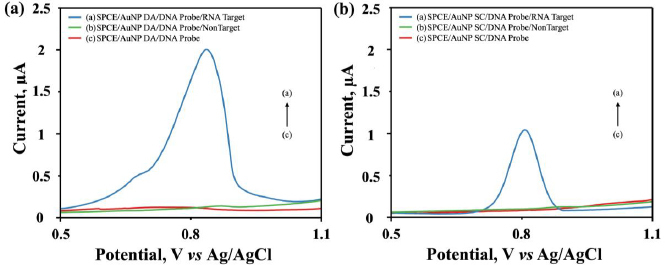
The difference of guanine oxidation current peak in DPV voltammograms for the DNA probe-target RNA hybridization, DNA probe-non target hybridization, and DNA probe on the SPCE/AuNPs by (a) DC and (b) SC. The solution was 0.01 M PBS with a scan rate of 0.008 V s^-1^ in the potential range of -0.8 to 1.2 V with a scan rate of 0.008 V s^-1^, *E*_step_ 0.005 V, *E*_pulse_ 0.05 V, and *t*_pulse_ 0.05 s

Figure 4 shows the result of measuring the guanine oxidation signal for probe DNA and DNA probe-RNA target hybridization using DC (Figure 4a) and SC (Figure 4b) methods. The guanine oxidation peak on the carbon-based electrode is in the potential range of 0.9 to 1.29 V [34]. The results show that the guanine oxidation peak is in the potential range of 0.7 to 0.9 V. By substituting guanine bases in the DNA probe sequence with inosine, the DNA probe no longer shows guanine oxidation peaks. Although inosine has characteristics similar to guanine and hybridizes with cytosine, it does not provide an oxidation signal response in the potential region. Therefore, only a guanine oxidation signal was detected during the hybridization of DNA probe-target RNA.

Hybridization with target RNA produced a higher peak current in the DC method (2.251 μA) compared to SC (1.052 μA), with each probe DNA concentration of 0.5 μg mL^*-1*^ and target RNA 1 μg mL^*-1*^. The selectivity of the DNA probe immobilized on SPCE/AuNP when hybridized with non-target RNA showed no current response, similar to the signal observed for the SPCE/AuNP/DNA probe as presented in Figure 4 (curve b). This showed that the biosensors could detect the target RNA from SARS-CoV-2.

### Optimization parameter biosensor using Box-Behnken experimental design

Optimization is improving the performance of a system, process, or product to obtain maximum benefits. BBD is a statistical method for determining which factors are significant to an outcome with minimum experimental replication. In this study, the three factors used were DNA probe concentration (*X*_1_), DNA probe immobilization time (*X*_2_), and DNA probe-target RNA hybridization time (*X*_3_). Each factor varies at three different levels, ranging from lowest (-1) to middle (0) and highest (+1). Subsequently, the current response measurement results are processed using the Minitab program [47].

In this study, a significance test was conducted using analysis of variance (ANOVA) to determine the existence of the influence of various variables tested. Significance is the magnitude of the probability or opportunity to make an error in deciding. The *ɑ*-value (significance level) was determined to indicate the allowable error, namely the 1-confidence level. The confidence level used was 95 %, corresponding to a significance level of *ɑ* = 0.05. A larger *p*-value (approaching 1/*p* > 0.05) showed that the factor did not have a significant influence on performance (*Y*). Meanwhile, a small *p*-value (close to zero, *p* < 0.05) significantly influences the response at the 95 % probability level. Based on the analysis, DNA probe concentration (*P* = 0.01) and DNA probe immobilization time (*P* = 0.007) were significant factors.

Lack of Fit (LOF) is a deviation or uncertainty in the first-order linear model. The P_lack of the fit_ value obtained was 0.208 based on the statistical analysis conducted using ANOVA, which means that the LOF was not significant, indicating that the linear model used was appropriate. Based on the regression equation obtained, decreased and increased responses were shown by factors with negative and positive values, respectively. Hence, the optimum experimental conditions obtained through BBD were 0.5 μg mL^-1^ for DNA probe concentration, 22 minutes for DNA probe immobilization time, and 12 minutes for DNA probe-target RNA hybridization time.

### Calibration curves of biosensor

**Figure 5.**
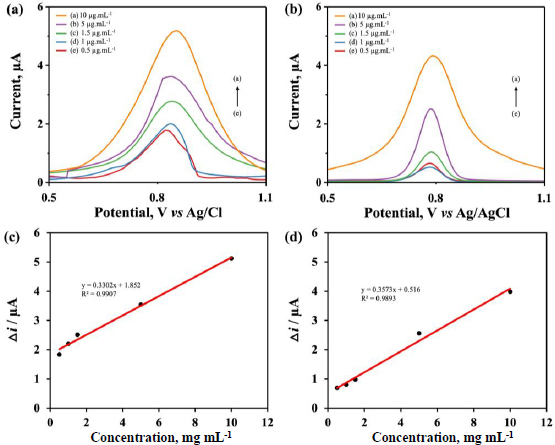
DPV voltammogram of guanine oxidation against variations in synthetic target RNA concentration on SPCE/AuNPs by (a) DC and (b) SC. Calibration curve of guanine oxidation signal against variation in target RNA concentration (0.5; 1; 1.5; 5; 10 μg mL^-1^) on SPCE/AuNP by (c) DC and (d) SC, measured using DPV with a scan rate of 0.008 V.s^-1^ over a potential range of -0.8 to +1.2 V in 0.01 M PBS solution

The response to variations in the concentration of synthetic target RNA was measured under optimum conditions using synthetic target RNA concentrations (0.5; 1.0, 1.5, 5.0 and 10.0 μg mL^-1^). The measurement was carried out in buffer solution using the DPV method over a potential range of -0.8 to +1.2 V with a scan rate of 0.008 V s^-1^. Based on Figures 5a and 5b, the concentration of the target RNA increased along with the peak current of the guanine oxidation signal for both electrodes modified by the DC and SC methods. Subsequently, linearity was determined to understand the relationship between the analysed synthetic target RNA concentration and the peak current of guanine oxidation produced (Figure 5c and 5d). The results showed that the average current response of target RNA with SPCE/AuNPs using DC was higher than SC across measurable concentration variations compared to the SC method. However, the voltammogram from the DC method showed lines with more movement or noise compared to SC. This was due to the inability to create a homogeneous distribution of Au on the SPCE surface compared to SC.

Furthermore, a T-test was used to compare the two methods. The null hypothesis (Ho) stated that there was no significant difference between the two methods, while the alternative hypothesis (Ha) reported a substantial variation. T-test giving results giving *t*_count_ of 6.992 and *t*_table_ of 2.447. This showed that the hypothesis being tested by Ho was rejected and the Ha was accepted, meaning that the results of SARS-CoV-2 RNA detection using SPCE/AuNPs via DC were significantly different from the SPCE/AuNPs modified via SC.

The linearity equation for the DC method in Figure 5c obtained the equation *y* = 0.3302*x* + 1.852 with *r*^2^ = 0.9907, while SC in Figure 5d gave the equation *y* = 0.357*x* + 0.516 with *r*^2^ = 0.9893. The slope values showed two equations, with DC electrochemically having higher sensitivity than SC. The limit of detection (LoD) was found to be 0.166 μg mL^-1^ and the limit of quantification (LoQ) at 0.505 μg mL^-1^ for DC, while SC had 0.694 and 2.105 μg mL^-1^, respectively. A total of six repeated measurements at one concentration of synthetic target RNA produced a precision of 99.7 % for DC and 99.4 % for SC, with accuracy rates of 94.4 % and 80.8 %, respectively. LoD and LoQ of SPCE/AuNPs modified using the DC method were lower compared to SC. Based on these results, the DC method was considered more sensitive despite SC having cleaner voltammograms with less noise. Therefore, the modification of SPCE/AuNPs using DC outperformed SC for detecting SARS-CoV-2 RNA. A comparison of SARS-CoV-2 detection using electrochemical biosensors in this study with other studies can be seen in Table 2.

**Table 2.** Research on electrochemical biosensors to detect SARS-CoV-2 using various electrode modification techniques

Electrode	Modification technique	LoD	Reference
Graphene electrode/AuNPs	Electrodeposition	6.9 copy μL^-1^	[37]
SPCE/AuNPs	Electrodeposition	1.0 copy μL^-1^	[38]
Thin-film/AuNPs	Electrodeposition	111 fM	[39]
SPCE/graphene	Electrodeposition	20 μg mL^-1^	[40]
SPCE/AuNPs	Drop casting	79.97 ng mL^-1^	[20]
SPCE/nano black carbon	Drop casting	8 ng mL^-1^	[22]
SPCE/Cu_2_O nanotubes	Spin coating	0.04 fg mL^-1^	[41]
SPCE/AuNPs	Drop casting and spray coating	DC: 0.166 μg mL^-1^, SC: 0.694 μg mL^-1^	This work

## Conclusions

This study highlights the successful modification of SPCE with AuNPs using drop casting (DC) and spray coating (SC) methods to enhance biosensor performance for SARS-CoV-2 RNA detection. The DC method achieved a higher density of AuNPs on the electrode surface, leading to improved electron transfer efficiency and a greater increase in peak current response compared to the SC method. In contrast, the SC method provided a more uniform distribution of AuNPs, albeit with a lower peak current response. Electrochemical characterizations using DPV and EIS confirmed improved conductivity and reduced resistance for both methods, with the DC method exhibiting higher sensitivity (LoD = 0.166 μg mL^-1^) compared to SC (LoD = 0.694 μg mL^-1^). Although the DC method offered greater sensitivity and a lower detection limit, the SC method produced cleaner, less noisy voltammograms. Both approaches demonstrated high selectivity, as non-target RNA hybridization did not produce a detectable signal. Overall, the DC method is more suitable for applications demanding high sensitivity, while the SC method is advantageous for achieving uniform nanoparticle coverage. This study also demonstrates the potential of SPCE/AuNP-based biosensors in clinical diagnostics and provides a solid foundation for further development in nucleic acid detection technologies.

## References

[1] Hartati Y.W. Gaffar S. Alfiani D. Pratomo U. Sofiatin Y. Subroto T. A voltammetric immunosensor based on gold nanoparticle - Anti-ENaC bioconjugate for the detection of epithelial sodium channel (ENaC) protein as a biomarker of hypertension. Sensing and Bio-Sensing Research 29 (2020) 100343. https://doi.org/10.1016/j.sbsr.2020.100343. 10.1016/j.sbsr.2020.100343

[2] Khater M. de la Escosura-Muñiz A. Quesada-González D. Merkoçi A. Electrochemical detection of plant virus using gold nanoparticle-modified electrodes. Analytica Chimica Acta 1046 (2019) 123-131. https://doi.org/10.1016/j.aca.2018.09.031. 10.1016/j.aca.2018.09.031 30482289

[3] Kivrak E. Pauzaite T. Copeland N.A. Hardy J.G. Kara P. Firlak M. Yardimci A.I. Yilmaz S. Palaz F. Ozsoz M. Detection of CRISPR-Cas9-Mediated Mutations Using a Carbon Nanotube-Modified Electrochemical Genosensor. Biosensors 11 (2021) 17. https://doi.org/10.3390/bios11010017. 10.3390/bios11010017 33429883 PMC7827051

[4] Davis D. Guo X. Musavi L. Lin C.S. Chen S.H. Wu V.C.H. Gold nanoparticle-modified carbon electrode biosensor for the detection of listeria monocytogenes. Industrial Biotechnology 9 (2013) 31-36. https://doi.org/10.1089/ind.2012.0033. 10.1089/ind.2012.0033

[5] Farghali R.A. Ahmed R.A. Gold nanoparticles-modified screen-printed carbon electrode for voltammetric determination of sildenafil citrate (Viagra) in pure form, biological and pharmaceutical formulations. International Journal of Electrochemical Science 10 (2015) 1494-1505. https://doi.org/10.1016/s1452-3981(23)05088-5. 10.1016/s1452-3981(23)05088-5

[6] Satriana N.P. Gaffar S. Subroto T. Hartati Y.W. Label-based and label-free electrochemical DNA biosensors for the detection of viruses: A review. Current Topics in Electrochemistry 23 (2021) 117-127. https://doi.org/10.31300/ctec.23.2021.117-127. 10.31300/ctec.23.2021.117-127

[7] Hartati Y.W. Nurdjanah D. Wyantuti S. Anggraeni A. Gaffar S. Gold nanoparticles modified screen-printed immunosensor for cancer biomarker HER2 determination based on anti HER2 bioconjugates. AIP Conference Proceedings 2049 (2018) 020051. https://doi.org/10.1063/1.5082456. 10.1063/1.5082456

[8] Ali M.Y. Abdulrahman H.B. Ting W.T. Howlader M.M.R. Green synthesized gold nanoparticles and CuO-based nonenzymatic sensor for saliva glucose monitoring. RSC Advances 14 (2024) 577-588. https://doi.org/10.1039/d3ra05644a. 10.1039/d3ra05644a 38173614 PMC10758929

[9] Fotovvati B. Namdari N. Dehghanghadikolaei A. On coating techniques for surface protection: A review. Journal of Manufacturing and Materials Processing 3 (2019) 28. https://doi.org/10.3390/jmmp3010028. 10.3390/jmmp3010028

[10] Anshori I. Althof R.R. Rizalputri L.N. Ariasena E. Handayani M. Pradana A. Akbar M.R. Syamsunarno M.R.A.A. Purwidyantri A. Prabowo B.A. Annas M.S. Munawar H. Yuliarto B. Gold Nanospikes Formation on Screen-Printed Carbon Electrode through Electrodeposition Method for Non-Enzymatic Electrochemical Sensor. Metals 12 (2022) 2116. https://doi.org/10.3390/met12122116. 10.3390/met12122116

[11] Bernardo-Boongaling V.R.R. Serrano N. García-Guzmán J.J. Palacios-Santander J.M. Díaz-Cruz J.M. Screen-printed electrodes modified with green-synthesized gold nanoparticles for the electrochemical determination of aminothiols. Journal of Electroanalytical Chemistry 847 (2019) 113184. https://doi.org/10.1016/j.jelechem.2019.05.066. 10.1016/j.jelechem.2019.05.066

[12] Svigelj R. Zuliani I. Grazioli C. Dossi N. Toniolo R. An Effective Label-Free Electrochemical Aptasensor Based on Gold Nanoparticles for Gluten Detection. Nanomaterials 12 (2022) 987. https://doi.org/10.3390/nano12060987. 10.3390/nano12060987 35335800 PMC8953296

[13] Xie K.X. Liu C. Liu Q. Xiao X.X. Li Z. Li M.F. Multiarchitecture-Based Plasmonic-Coupled Emission Employing Gold Nanoparticles: An Efficient Fluorescence Modulation and Biosensing Platform. Langmuir 37 (2021) 11880-11886. https://doi.org/10.1021/acs.langmuir.1c01965. 10.1021/acs.langmuir.1c01965 34592818

[14] Karim M.N. Lee J.E. Lee H.J. Amperometric detection of catechol using tyrosinase modified electrodes enhanced by the layer-by-layer assembly of gold nanocubes and polyelectrolytes. Biosensors and Bioelectronics 61 (2014) 147-151. https://doi.org/10.1016/j.bios.2014.05.011. 10.1016/j.bios.2014.05.011 24874658

[15] Gusmão R. López-Puente V. Yate L. Pastoriza-Santos I. Pérez-Juste J. González-Romero E. Screen-printed carbon electrodes doped with TiO2-Au nanocomposites with improved electrocatalytic performance. Materials Today Communications 11 (2017) 11-17. https://doi.org/10.1016/j.mtcomm.2017.02.003. 10.1016/j.mtcomm.2017.02.003

[16] Syafira R.S. Devi M.J. Gaffar Irkham S. Kurnia I. Arnafia W. Einaga Y. Syakir N. Noviyanti A.R. Hartati Y.W. Hydroxyapatite-Gold Modified Screen-Printed Carbon Electrode for Selective SARS-CoV-2 Antibody Immunosensor. ACS Applied Bio Materials 7 (2024) 950-960. https://doi.org/10.1021/acsabm.3c00953. 10.1021/acsabm.3c00953 38303668

[17] Mayousse C. Celle C. Moreau E. Mainguet J.F. Carella A. Simonato J.P. Improvements in purification of silver nanowires by decantation and fabrication of flexible transparent electrodes. Application to capacitive touch sensors. Nanotechnology 24 (2013) 215501. https://doi.org/10.1088/0957-4484/24/21/215501. 10.1088/0957-4484/24/21/215501 23619480

[18] Boulanger N. Skrypnychuk V. Nordenström A. Moreno-Fernández G. Granados-Moreno M. Carriazo D. Mysyk R. Bracciale G. Bondavalli P. Talyzin A. V. Spray Deposition of Supercapacitor Electrodes using Environmentally Friendly Aqueous Activated Graphene and Activated Carbon Dispersions for Industrial Implementation. ChemElectroChem 8 (2021) 1349-1361. https://doi.org/10.1002/celc.202100235. 10.1002/celc.202100235

[19] Mahmoodi P. Rezayi M. Rasouli E. Avan A. Gholami M. Ghayour Mobarhan M. Karimi E. Alias Y. Early-stage cervical cancer diagnosis based on an ultra-sensitive electrochemical DNA nanobiosensor for HPV-18 detection in real samples. Journal of Nanobiotechnology 18 (2020) 11. https://doi.org/10.1186/s12951-020-0577-9. 10.1186/s12951-020-0577-9 31931815 PMC6956556

[20] Sari A.K. Hartati Y.W. Gaffar S. Anshori I. Hidayat D. Wiraswati H.L. The optimization of an electrochemical aptasensor to detect RBD protein S SARS-CoV-2 as a biomarker of COVID-19 using screen-printed carbon electrode/AuNP. Journal of Electrochemical Science and Engineering 12 (2022) 219-235. https://doi.org/10.5599/jese.1206. 10.5599/jese.1206

[21] Zakiyyah S.N. Irkham Einaga Y. Gultom S.N. Fauzia R.P. Kadja G.T.M. Gaffar S. Ozsoz H. Hartati Y.W. Green Synthesis of Ceria Nanoparticles from Cassava Tubers for Electrochemical Aptasensor Detection of SARS-CoV-2 on a Screen-Printed Carbon Electrode. ACS Applied Bio Materials 7 (2024) 2488-2498. https://doi.org/10.1021/acsabm.4c00088. 10.1021/acsabm.4c00088 38577953

[22] Raziq A. Kidakova A. Boroznjak R. Reut J. Öpik A. Syritski V. Development of a portable MIP-based electrochemical sensor for detection of SARS-CoV-2 antigen. Biosensors and Bioelectronics 178 (2021) 113029. https://doi.org/10.1016/j.bios.2021.113029. 10.1016/j.bios.2021.113029 33515985 PMC7826012

[23] Drobysh M. Liustrovaite V. Kanetski Y. Brasiunas B. Zvirbliene A. Rimkute A. Gudas D. Kucinskaite-Kodze I. Simanavicius M. Ramanavicius S. Slibinskas R. Ciplys E. Plikusiene I. Ramanavicius A. Electrochemical biosensing based comparative study of monoclonal antibodies against SARS-CoV-2 nucleocapsid protein. Science of The Total Environment 908 (2024) 168154. https://doi.org/10.1016/j.scitotenv.2023.168154. 10.1016/j.scitotenv.2023.168154 37923263

[24] Devarakonda S. Singh R. Bhardwaj J. Jang J. Cost-effective and handmade paper-based immunosensing device for electrochemical detection of influenza virus. Sensors 17 (2017) 2597. https://doi.org/10.3390/s17112597. 10.3390/s17112597 29137115 PMC5713655

[25] Chomoucka J. Prasek J. Businova P. Trnkova L. Drbohlavova J. Pekarek J. Hrdy R. Hubalek J. Novel electrochemical biosensor for simultaneous detection of adenine and guanine based on Cu2O nanoparticles. Procedia Engineering 47 (2012) 702-705. https://doi.org/10.1016/j.proeng.2012.09.244. 10.1016/j.proeng.2012.09.244

[26] Say M.G. Brooke R. Edberg J. Grimoldi A. Belaineh D. Engquist I. Berggren M. Spray-coated paper supercapacitors. npj Flexible Electronics 4 (2020) 1-7. https://doi.org/10.1038/s41528-020-0079-8. 10.1038/s41528-020-0079-8

[27] Li X. Geng M. Peng Y. Meng L. Lu S. Molecular immune pathogenesis and diagnosis of COVID-19. Journal of Pharmaceutical Analysis 10 (2020) 102-108. https://doi.org/10.1016/j.jpha.2020.03.001. 10.1016/j.jpha.2020.03.001 32282863 PMC7104082

[28] Astuti I. Ysrafil. Severe Acute Respiratory Syndrome Coronavirus 2 (SARS-CoV-2): An overview of viral structure and host response. Diabetes and Metabolic Syndrome: Clinical Research and Reviews 14 (2020) 407-412. https://doi.org/10.1016/j.dsx.2020.04.020. 10.1016/j.dsx.2020.04.020 PMC7165108 32335367

[29] Rashid J.I.A. Yusof N.A. The strategies of DNA immobilization and hybridization detection mechanism in the construction of electrochemical DNA sensor: A review. Sensing and Bio-Sensing Research 16 (2017) 19-31. https://doi.org/10.1016/j.sbsr.2017.09.001. 10.1016/j.sbsr.2017.09.001

[30] Peng Y. Pan Y. Sun Z. Li J. Yi Y. Yang J. Li G. An electrochemical biosensor for sensitive analysis of the SARS-CoV-2 RNA. Biosensors and Bioelectronics 186 (2021) 113309. https://doi.org/10.1016/j.bios.2021.113309. 10.1016/j.bios.2021.113309 33984795 PMC8107000

[31] Kashefi-Kheyrabadi L. Nguyen H.V. Go A. Baek C. Jang N. Lee J.M. Cho N.H. Min J. Lee M.H. Rapid, multiplexed, and nucleic acid amplification-free detection of SARS-CoV-2 RNA using an electrochemical biosensor. Biosensors and Bioelectronics 195 (2022) 113649. https://doi.org/10.1016/j.bios.2021.113649. 10.1016/j.bios.2021.113649 34555637 PMC8447555

[32] Kerman K. Morita Y. Takamura Y. Tamiya E. Label-free electrochemical detection of DNA hybridization on gold electrode. Electrochemistry Communications 5 (2003) 887-891. https://doi.org/10.1016/j.elecom.2003.08.013. 10.1016/j.elecom.2003.08.013

[33] Hartati Y.W. Wyantuti S. Lutfi Firdaus M. Auliany N. Surbakti R. Gaffar S. A rapid and sensitive diagnosis of typhoid fever based on nested PCR-Voltammetric DNA biosensor using flagellin gene fragment. Indonesian Journal of Chemistry 16 (2016) 87-91. https://doi.org/10.22146/ijc.21182. 10.22146/ijc.21182

[34] Hartati Y.W. Suryani A.A. Agustina M. Gaffar S. Anggraeni A. A Gold Nanoparticle-DNA Bioconjugate-Based Electrochemical Biosensor for Detection of Sus scrofa mtDNA in Raw and Processed Meat. Food Analytical Methods 12 (2019) 2591-2600. https://doi.org/10.1007/s12161-019-01593-6. 10.1007/s12161-019-01593-6

[35] Hartati Y.W. Irkham I. Sumiati I. Wyantuti S. Gaffar S. Zakiyyah S.N. Zein M.I.H.L. Ozsoz M. The Optimization of a Label-Free Electrochemical DNA Biosensor for Detection of Sus scrofa mtDNA as Food Adulterations. Biosensors 13 (2023) 657. https://doi.org/10.3390/bios13060657. 10.3390/bios13060657 37367022 PMC10296283

[36] Tripathy S. Singh S.G. Label-Free Electrochemical Detection of DNA Hybridization: A Method for COVID-19 Diagnosis. Transactions of the Indian National Academy of Engineering 5 (2020) 205-209. https://doi.org/10.1007/s41403-020-00103-z. 10.1007/s41403-020-00103-z 38624377 PMC7247285

[37] Alafeef M. Dighe K. Moitra P. Pan D. Rapid, Ultrasensitive, and Quantitative Detection of SARS-CoV-2 Using Antisense Oligonucleotides Directed Electrochemical Biosensor Chip. ACS Nano 14 (2020) 17028-17045. https://doi.org/10.1021/acsnano.0c06392. 10.1021/acsnano.0c06392 33079516

[38] Chaibun T. Puenpa J. Ngamdee T. Boonapatcharoen N. Athamanolap P. O’Mullane A.P. Vongpunsawad S. Poovorawan Y. Lee S.Y. Lertanantawong B. Rapid electrochemical detection of coronavirus SARS-CoV-2. Nature Communications 12 (2021) 802. https://doi.org/10.1038/s41467-021-21121-7. 10.1038/s41467-021-21121-7 PMC7864991 33547323

[39] Fabiani L. Saroglia M. Galatà G. De Santis R. Fillo S. Luca V. Faggioni G. D’Amore N. Regalbuto E. Salvatori P. Terova G. Moscone D. Lista F. Arduini F. Magnetic beads combined with carbon black-based screen-printed electrodes for COVID-19: A reliable and miniaturized electrochemical immunosensor for SARS-CoV-2 detection in saliva. Biosensors and Bioelectronics 171 (2021) 112686. https://doi.org/10.1016/j.bios.2020.112686. 10.1016/j.bios.2020.112686 33086175 PMC7833515

[40] Mojsoska B. Larsen S. Olsen D.A. Madsen J.S. Brandslund I. Alatraktchi F.A. Rapid SARS-CoV-2 detection using electrochemical immunosensor. Sensors 21 (2021) 390. https://doi.org/10.3390/s21020390. 10.3390/s21020390 33429915 PMC7827295

[41] Rahmati Z. Roushani M. Hosseini H. Choobin H. Electrochemical immunosensor with Cu2O nanocube coating for detection of SARS-CoV-2 spike protein. Microchimica Acta 188 (2021) 105. https://doi.org/10.1007/s00604-021-04762-9. 10.1007/s00604-021-04762-9 33651173 PMC7921825

[42] Emami M. Shamsipur M. Saber R. Irajirad R. An electrochemical immunosensor for detection of a breast cancer biomarker based on antiHER2-iron oxide nanoparticle bioconjugates. Analyst 139 (2014) 2858-2866. https://doi.org/10.1039/c4an00183d. 10.1039/c4an00183d 24752767

[43] Zarei S.S. Soleimanian-Zad S. Ensafi A.A. An impedimetric aptasensor for Shigella dysenteriae using a gold nanoparticle-modified glassy carbon electrode. Microchimica Acta 185 (2018) 538. https://doi.org/10.1007/s00604-018-3075-0. 10.1007/s00604-018-3075-0 30413894

[44] Liu B. Liu J. Freezing Directed Construction of Bio/Nano Interfaces: Reagentless Conjugation, Denser Spherical Nucleic Acids, and Better Nanoflares. Journal of the American Chemical Society 139 (2017) 9471-9474. https://doi.org/10.1021/jacs.7b04885. 10.1021/jacs.7b04885 28661156

[45] Liu B. Liu J. Methods for preparing DNA-functionalized gold nanoparticles, a key reagent of bioanalytical chemistry. Analytical Methods 9 (2017) 2633-2643. https://doi.org/10.1039/c7ay00368d. 10.1039/c7ay00368d

[46] Jain P.K. Qian W. El-Sayed M.A. Ultrafast cooling of photoexcited electrons in gold nanoparticle-thiolated DNA conjugates involves the dissociation of the gold-thiol bond. Journal of the American Chemical Society 128 (2006) 2426-2433. https://doi.org/10.1021/ja056769z. 10.1021/ja056769z 16478198

[47] Gong Q. Yang H. Dong Y. Zhang W. A sensitive impedimetric DNA biosensor for the determination of the HIV gene based on electrochemically reduced graphene oxide. Analytical Methods 7 (2015) 2554-2562. https://doi.org/10.1039/c5ay00111k 10.1039/c5ay00111k

